# Crystal structure and mol­ecular docking study of diethyl 2,2′-({[(1*E*,1′*E*)-(hydrazine-1,2-diyl­idene)bis­(methanylyl­idene)]bis­(4,1-phenyl­ene)}bis­(­oxy))di­acetate

**DOI:** 10.1107/S205698902101344X

**Published:** 2022-01-01

**Authors:** Said Daoui, Sevgi Kansiz, Feyzi Alkim Aktas, Necmi Dege, Eiad Saif, Noureddine Benchat, Khalid Karrouchi

**Affiliations:** a Laboratory of Applied Chemistry and Environment (LCAE), Faculty of Sciences, Mohammed I University, 60000 Oujda, Morocco; b Samsun University, Faculty of Engineering, Department of Fundamental Sciences, 55420, Samsun, Turkey; c Samsun University, Faculty of Engineering, Biomedical Engineering, Samsun, 55420, Turkey; d Ondokuz Mayıs University, Faculty of Arts and Sciences, Department of Physics, 55139, Samsun, Turkey; e Department of Computer and Electronic Engineering Technology, Sanaa Community College, Sanaa, Yemen; f Department of Electrical and Electronic Engineering, Faculty of Engineering, Ondokuz Mayıs University, 55139, Samsun, Turkey; g Laboratory of Analytical Chemistry and Bromatology, Faculty of Medicine and Pharmacy, Mohammed V University in Rabat, Morocco

**Keywords:** crystal structure, Schiff base, hydrazine, mol­ecular docking

## Abstract

The title Schiff base, C_22_H_24_N_2_O_6_, adopts an *E* configuration. The mol­ecule is planar, the mean planes of the phenyl ring system (r.m.s deviation = 0.0059 Å) forms a dihedral angle of 0.96 (4)° with the mean plane of the phenyl ring moiety (r.m.s deviation = 0.0076 Å). In the crystal, mol­ecules are linked by weak inter­molecular C—H⋯O and C—H⋯N hydrogen bonds into chains extending along the *c*-axis and *b*-axis directions, respectively. A mol­ecular docking study between the title mol­ecule and 5-HT2C, which is a G protein receptor and ligand-gated ion channels found in nervous systems (PDB ID: 6BQH) was executed. The experiment shows that it is a good potential agent because of its affinity and ability to stick to the active sites of the receptor.

## Abstract 2

The title Schiff base adopts an *E* configuration. The mol­ecular structure is stabilized by an C—H⋯O and C—H⋯N hydrogen bonds.

## Chemical context

Compounds with an azomethine group (–C=N–) are known as Schiff bases, which are usually synthesized from the condensation of active carbonyl groups and primary amines (Yang *et al.*, 2001 ▸). Furthermore, these derivatives represent an important class of organic compounds, especially in the medicinal and pharmaceutical fields (Murtaza *et al.*, 2014 ▸). It is well known from the literature that Schiff bases display excellent biological properties, such as anti­oxidant and analgesic (Karrouchi *et al.*, 2016 ▸), anti­bacterial and cytotoxic (Maaref *et al.*, 2020 ▸), anti­diabetic (Karrouchi *et al.*, 2022 ▸) and anti-inflammatory activities (Rana *et al.*, 2012 ▸). These deriv­atives are also used as corrosion inhibitors, which relies on their ability to spontaneously form a monolayer on the surface being protected (El Arrouji *et al.*, 2020 ▸). In this study, the title compound, diethyl 2,2′-({[(1*E*,1′*E*)-(hydrazine-1,2-diyl­idene)bis(methanylyl­idene)]bis­(4,1-phenyl­ene)}bis­(­oxy))di­acetate, was characterized by single crystal X-ray and studied by Hirshfeld surface analysis. 
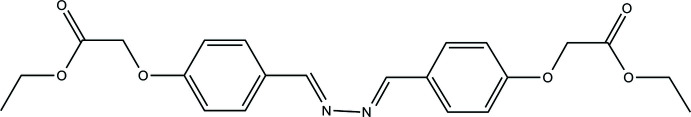


## Structural commentary

**Figure 1:**
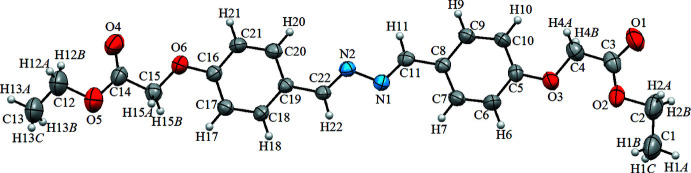
The mol­ecular structure of the title compound, with atom labelling. Displacement ellipsoids are drawn at the 40% probability level.

The mol­ecular structure of the title compound is illustrated in Fig. 1 ▸. The asymmetric unit contains one independent mol­ecule, which is planar, the mean plane of the C5–C10 phenyl ring (r.m.s deviation = 0.006 Å) forms a dihedral angle of 0.96 (4)° with the mean plane of the C16–C20 phenyl ring (r.m.s deviation = 0.008 Å). The C3—O1 and C14—O4 bond lengths in the mol­ecule are 1.213 (8) and 1.212 (8) Å, respectively, while the C11—N1 and C22—N2 bond lengths are 1.274 (7) and 1.275 (7) Å, respectively (Table 1 ▸). These results suggest a double-bond character for the C=O and C=N bonds. The N1—N2 bond distance, 1.419 (7) Å, is compatible with 1.411 Å (Manawar *et al.*, 2019 ▸; Kansiz *et al.*, 2021 ▸). These results suggest a single bond character for N—N, as expected from hydrazine structures. The exocyclic angles C4—C3—O2 [115.4 (6)°], O1—C3—O2 [125.4 (8)°], C15—C14—O4 [125.5 (7)°] and C15—C14—O5 [111.9 (6)°] deviate significantly from the normal value of 120°; this may be due to steric repulsion (H4*A*⋯H10 = 2.22 Å and H15*B*⋯H17 = 2.32 Å). Bond lengths and angles are within normal ranges and are comparable to those observed in related structures (see *Database survey* section).

## Supra­molecular features

**Figure 2:**
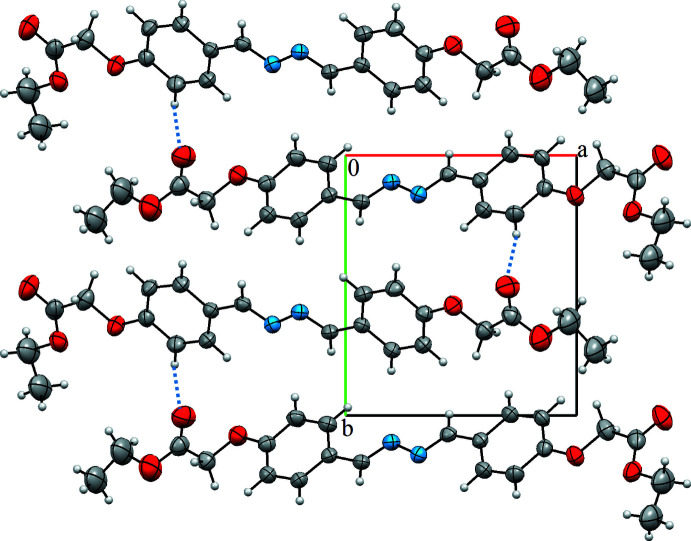
The crystal packing of the title compound with the inter­molecular C—H⋯O hydrogen bonds shown as dashed lines.

In the crystal, there are two inter­molecular hydrogen bonds. The C6—H6⋯O4^i^ hydrogen bond links the mol­ecules to each other along the *c*-axis direction while the C4—H4*B*⋯N1^ii^ hydrogen bond links the mol­ecules to each other along the *b*-axis direction (symmetry codes as in Table 1 ▸). A view of the crystal packing is shown in Fig. 2 ▸.

## Database survey

A search of the Cambridge Structural Database (CSD, version 5.42, update of May 2021; Groom *et al.*, 2016 ▸) for the ethyl 2-(*p*-tol­yloxy)acetate skeleton revealed seven similar compounds, *viz.*: ethyl 4-[1-(4-bromo­phen­yl)-3-methyl-5-oxo-4,5-di­hydro-1*H*-1,2,4-triazol-4-yl­imino­meth­yl]phen­oxy­acetate (EKEYEY; Thamotharan *et al.*, 2003 ▸), di[3-fluoro-6-meth­oxy-4-(eth­oxy­carbonyl­meth­oxy)benz­yl] ether (HIGLEP; Wallner *et al.*, 2007 ▸), ethyl (2-fluoro-4-hy­droxy­methyl-5-meth­oxy­phen­oxy)acetate (HIGLIT; Wallner *et al.*, 2007 ▸), diethyl 3,3-bis­{3-[4-(2-eth­oxy-2-oxoeth­oxy)-3-meth­oxy­phen­yl]acrylo­yl}penta­nedioate (JUMJEI; Xu *et al.*, 2015 ▸), ethyl (4-{3-[2,4-bis­(2-eth­oxy-2-oxoeth­oxy)phen­yl]-3-oxoprop-1-en-1-yl}phen­oxy)acetate (PIXWAW; Liu, 2014 ▸), ethyl [(2-oxo-2*H*-chromen-7-yl)­oxy]acetate (WIHDEY; Fun *et al.*, 2013 ▸) and ethyl {4-[(*E*)-2-(3,4,5-tri­meth­oxy­phen­yl)vin­yl]phen­oxy}acetate (XEWZIJ; Baolin *et al.*, 2007 ▸). In EKEYEY, the eth­oxy­carbonyl­meth­oxy group is oriented at an angle of 29.42 (15)° with respect to the mean plane of the benzene ring. The mean plane of the 2*H*-chromene ring system (O1/C1–C9, r.m.s deviation = 0.026 Å) forms a dihedral angle of 81.71 (6)° with the mean plane of ethyl 2-hy­droxy­acetate moiety (O1/N3/C9/C10, r.m.s deviation = 0.026 Å) in WIHDEY. This dihedral angle for the title compound is smaller than in both EKEYEY and WIHDEY with a value of 4.38 (8)°. The C10—C11 bond distance of 1.516 (2) Å in WIHDEY, corresponding to a single bond, is slightly longer than observed for the title compound [C3—C4 = 1.498 (10) Å]. This bond length is also longer than in XEWZIJ [C18–C19 = 1.493 (3) Å; Baolin *et al.*, 2007 ▸)].

## Mol­ecular docking study

**Figure 3:**
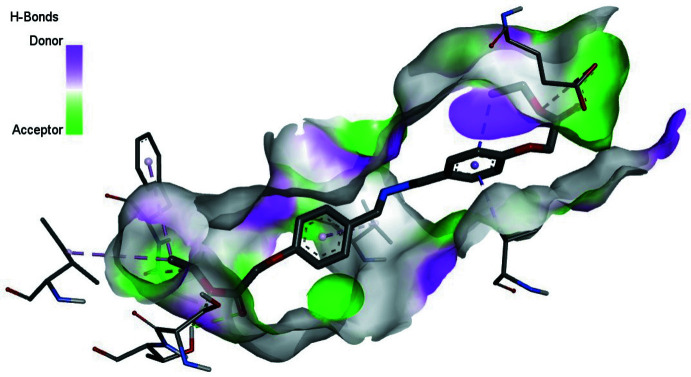
Three-dimensional visual of the inter­molecular inter­actions for the best binding pose of the title compound docking with 6BQH.

**Figure 4:**
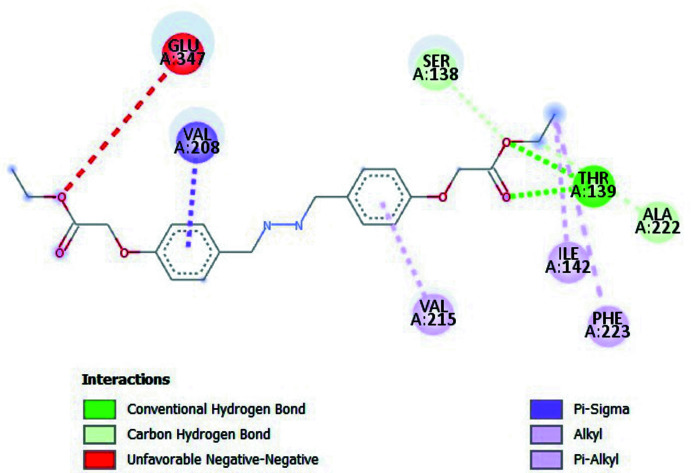
Two-dimensional visual of the inter­molecular inter­actions for the best binding pose of the title compound docking with 6BQH.

**Figure 5:**
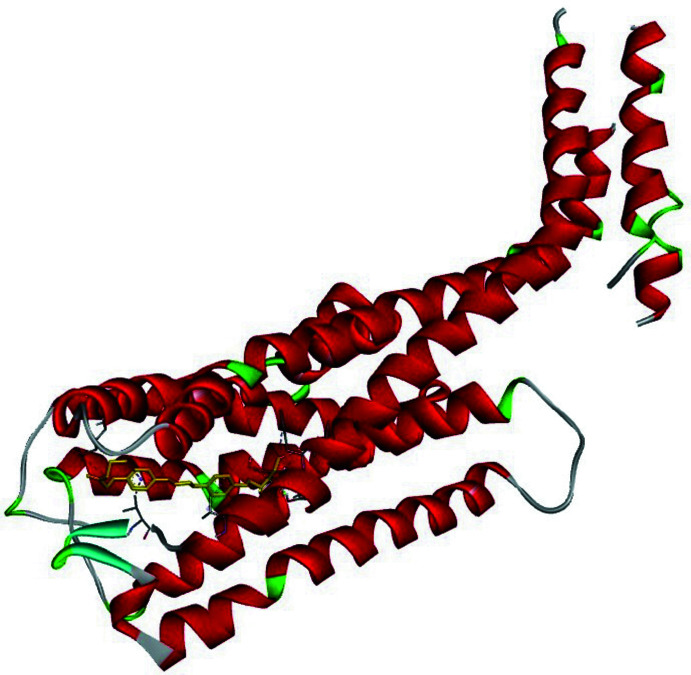
Three-dimensional conformation of the title compound with 6BQH.

Mol­ecular docking is a substantial process for finding the inter­actions between small mol­ecules and macromolecules. Inter­molecular bonds that occur between ligand and receptor are indicated by mol­ecular docking. In this study, *AutoDockVina* (Trott & Olson, 2010 ▸) was used for predictive binding sites between the title mol­ecule and the 5-HT2C receptor (Peng *et al.*, 2018 ▸). 6BQH is a serotonin receptor, which can be efficient for designing drugs to treat ailments such as anxiety, aggression, sleep disorders, and other psychological diseases. The three-dimensional structure of 6BQH was taken from the Protein Data Bank (PDB). Before the docking calculations, the receptor must be prepared for efficient insertion. For this reason, all water and ligand mol­ecules were cleared on receptor active sites. According to these active sites, grid box dimensions were defined as 100 x 80 x 110 Å. In addition, –*x*, *y*, *z* centres were adjusted to be −40.569, 33.142, 45.392, respectively, and then the 5-HT2C receptor was saved in PDBQT format for the calculations. In the next step, rotatable angles for the coupling structure were determined and recorded in PDBQT format. *Discovery Studio Visualizer* (Biovia, 2017 ▸) was used for observations and preparations. All docking calculations were calculated with *AutoDockVina*. Twenty variable links were decided by *AutoDockVina* for the ligands connected to the receptor of the protein. The best affinity energy was observed in the first calculation, which is −6.2 kcal mol^−1^. The bonding type of inter­action is represented in Fig. 3 ▸. The 2D and 3D visuals of the inter­molecular inter­actions for the best binding pose of the title compound docked into macromolecule 6BQH can be seen in Fig. 4 ▸. In addition, docking conformation can be seen in Fig. 5 ▸. Consequently, the title compound could be a possible mol­ecule for drug design to treat psychological disorders, because its ability is suitable to stick to active sites of the receptor.

## Synthesis and crystallization

Hydrazine hydrate (0.013 g, 0.24 mmol) was added dropwise to a solution of ethyl 2-(4-formyl­phen­oxy)acetate (0.5 g, 0.48 mmol) in ethanol (20 ml), and the mixture was refluxed for 4 h. After cooling, the solvent was removed under reduced pressure, and the residue was purified by recrystallization from ethanol to afford single crystals (yield 80%).

## Refinement

Crystal data, data collection and structure refinement details are summarized in Table 2 ▸. C-bound H atoms were positioned geometrically and refined using a riding model with C—H = 0.93–0.97 Å and *U*_iso_(H) = 1.5*U*_eq_(C) for methyl H atoms, and *U*_iso_(H) = 1.2*U*_eq_(C) for all other H atoms. The crystal studied was refined as a two-component inversion twin, but the absolute structure was indeterminate.

## Supplementary Material

Crystal structure: contains datablock(s) I. DOI: 10.1107/S205698902101344X/zn2012sup1.cif

Structure factors: contains datablock(s) I. DOI: 10.1107/S205698902101344X/zn2012Isup2.hkl

Supporting information file. DOI: 10.1107/S205698902101344X/zn2012Isup3.cml

CCDC reference: 2129693

Additional supporting information:  crystallographic information; 3D view; checkCIF report

## Figures and Tables

**Table 1:** Hydrogen-bond geometry (Å, °)

*D*—H⋯*A*	*D*—H	H⋯*A*	*D*⋯*A*	*D*—H⋯*A*
C6—H6⋯O4^i^	0.93	2.57	3.483 (9)	169
C4—H4*B*⋯N1^ii^	0.97	2.69	3.618 (10)	161

Symmetry codes: (i) -x, y+{\script{1\over 2}}, -z-{\script{3\over 2}}; (ii) x-1, y, z.

**Table 2:** Experimental details

Crystal data	
Chemical formula	C_22_H_24_N_2_O_6_
*M*_r_	412.43
Crystal system, space group	Orthorhombic, *P*2_1_2_1_2_1_
Temperature (K)	296
*a*, *b*, *c* (Å)	8.1864 (4), 9.2061 (5), 27.7903 (18)
*V* (Å^3^)	2094.4 (2)
*Z*	4
Radiation type	Mo *K*α
μ (mm^−1^)	0.10
Crystal size (mm)	0.68 × 0.44 × 0.22

Data collection	
Diffractometer	Stoe IPDS 2
Absorption correction	Integration (*X-RED32*; Stoe & Cie, 2002 ▸)
*T*_min_, *T*_max_	0.945, 0.979
No. of measured, independent and observed [*I* > 2σ(*I*)] reflections	11156, 4091, 2453
*R*_int_	0.037
(sin θ/λ)_max_ (Å^−1^)	0.617

Refinement	
*R*[*F* ^2^ > 2σ(*F* ^2^)], *wR*(*F* ^2^), *S*	0.073, 0.246, 1.01
No. of reflections	4091
No. of parameters	254
No. of restraints	2
H-atom treatment	H-atom parameters constrained
Δρ_max_, Δρ_min_ (e Å^−3^)	0.50, −0.67
Absolute structure	Refined as an inversion twin, but the absolute structure was indeterminate
Absolute structure parameter	−1 (4)

Computer programs: *X-AREA* and *X-RED* (Stoe & Cie, 2002 ▸), *SHELXT2017/1* (Sheldrick, 2015*a* ▸), *SHELXL2017/1* (Sheldrick, 2015*b* ▸), *PLATON* (Spek, 2020 ▸) and *WinGX* (Farrugia, 2012 ▸).

## supplementary crystallographic information

### Crystal data

**Table:**

C_22_H_24_N_2_O_6_	*D*_x_ = 1.308 Mg m^−^^3^
*M**_r_* = 412.43	Mo *K*α radiation, λ = 0.71073 Å
Orthorhombic, *P*2_1_2_1_2_1_	Cell parameters from 9754 reflections
*a* = 8.1864 (4) Å	θ = 2.2–27.8°
*b* = 9.2061 (5) Å	µ = 0.10 mm^−^^1^
*c* = 27.7903 (18) Å	*T* = 296 K
*V* = 2094.4 (2) Å^3^	Prism, colorless
*Z* = 4	0.68 × 0.44 × 0.22 mm
*F*(000) = 872	

### Data collection

**Table:**

Stoe IPDS 2 diffractometer	4091 independent reflections
Radiation source: sealed X-ray tube, 12 x 0.4 mm long-fine focus	2453 reflections with *I* > 2σ(*I*)
Detector resolution: 6.67 pixels mm^-1^	*R*_int_ = 0.037
rotation method scans	θ_max_ = 26.0°, θ_min_ = 2.3°
Absorption correction: integration (X-RED32; Stoe & Cie, 2002)	*h* = −8→10
*T*_min_ = 0.945, *T*_max_ = 0.979	*k* = −11→11
11156 measured reflections	*l* = −27→34

### Refinement

**Table:**

Refinement on *F*^2^	Hydrogen site location: inferred from neighbouring sites
Least-squares matrix: full	H-atom parameters constrained
*R*[*F*^2^ > 2σ(*F*^2^)] = 0.073	*w* = 1/[σ^2^(*F*_o_^2^) + (0.1576*P*)^2^] where *P* = (*F*_o_^2^ + 2*F*_c_^2^)/3
*wR*(*F*^2^) = 0.246	(Δ/σ)_max_ < 0.001
*S* = 1.00	Δρ_max_ = 0.50 e Å^−^^3^
4091 reflections	Δρ_min_ = −0.67 e Å^−^^3^
254 parameters	Absolute structure: Refined as an inversion twin
2 restraints	Absolute structure parameter: −1 (4)

### Special details

**Table:**

Geometry. All esds (except the esd in the dihedral angle between two l.s. planes) are estimated using the full covariance matrix. The cell esds are taken into account individually in the estimation of esds in distances, angles and torsion angles; correlations between esds in cell parameters are only used when they are defined by crystal symmetry. An approximate (isotropic) treatment of cell esds is used for estimating esds involving l.s. planes.
Refinement. Refined as a 2-component inversion twin.

### Fractional atomic coordinates and isotropic or equivalent isotropic displacement parameters (Å^2^)

**Table:**

	*x*	*y*	*z*	*U*_iso_*/*U*_eq_	
O6	0.4659 (6)	−0.4221 (5)	−0.88303 (18)	0.0861 (14)	
O3	−0.9934 (5)	−0.3444 (5)	−0.61477 (18)	0.0815 (13)	
N2	−0.1980 (6)	−0.3968 (4)	−0.76468 (19)	0.0658 (12)	
O2	−1.2412 (6)	−0.2842 (6)	−0.5611 (2)	0.0968 (15)	
N1	−0.3161 (6)	−0.3494 (5)	−0.73078 (19)	0.0671 (13)	
O4	0.7008 (8)	−0.4943 (6)	−0.9435 (2)	0.122 (2)	
O5	0.8443 (9)	−0.2982 (8)	−0.9259 (3)	0.145 (2)	
O1	−1.3702 (7)	−0.4910 (7)	−0.5761 (2)	0.123 (2)	
C19	0.0707 (7)	−0.3464 (5)	−0.7945 (2)	0.0587 (13)	
C8	−0.5825 (7)	−0.4041 (5)	−0.7005 (2)	0.0590 (13)	
C3	−1.2583 (9)	−0.4068 (9)	−0.5824 (3)	0.090 (2)	
C5	−0.8617 (7)	−0.3742 (6)	−0.6434 (2)	0.0659 (15)	
C16	0.3406 (8)	−0.3906 (6)	−0.8529 (2)	0.0656 (15)	
C11	−0.4446 (7)	−0.4275 (5)	−0.7311 (2)	0.0618 (14)	
H11	−0.448969	−0.504718	−0.752671	0.074*	
C9	−0.7122 (7)	−0.5040 (6)	−0.7029 (2)	0.0689 (15)	
H9	−0.705537	−0.581369	−0.724270	0.083*	
C18	0.2009 (7)	−0.2516 (5)	−0.7937 (2)	0.0655 (15)	
H18	0.198036	−0.172160	−0.773028	0.079*	
C17	0.3358 (7)	−0.2717 (6)	−0.8230 (2)	0.0677 (15)	
H17	0.421932	−0.205814	−0.822424	0.081*	
C10	−0.8487 (7)	−0.4902 (6)	−0.6743 (2)	0.0677 (15)	
H10	−0.931717	−0.558927	−0.675966	0.081*	
C22	−0.0682 (7)	−0.3205 (5)	−0.7625 (2)	0.0617 (14)	
H22	−0.061751	−0.246143	−0.739922	0.074*	
C20	0.0776 (9)	−0.4663 (5)	−0.8255 (2)	0.0710 (16)	
H20	−0.009621	−0.530867	−0.827039	0.085*	
C6	−0.7324 (8)	−0.2740 (6)	−0.6404 (2)	0.0764 (17)	
H6	−0.739197	−0.196246	−0.619141	0.092*	
C21	0.2131 (8)	−0.4884 (6)	−0.8537 (2)	0.0756 (17)	
H21	0.218970	−0.569887	−0.873361	0.091*	
C4	−1.1264 (9)	−0.4449 (8)	−0.6175 (3)	0.0857 (19)	
H4A	−1.086821	−0.542135	−0.610821	0.103*	
H4B	−1.171001	−0.444297	−0.649865	0.103*	
C7	−0.5976 (8)	−0.2901 (6)	−0.6684 (2)	0.0711 (16)	
H7	−0.513326	−0.222775	−0.665958	0.085*	
C14	0.7176 (9)	−0.3829 (8)	−0.9207 (3)	0.0848 (19)	
C15	0.6016 (8)	−0.3258 (7)	−0.8849 (3)	0.0785 (17)	
H15A	0.653522	−0.320003	−0.853552	0.094*	
H15B	0.565809	−0.229246	−0.894006	0.094*	
C12	0.9676 (12)	−0.3616 (12)	−0.9585 (5)	0.145 (2)	
H12A	1.024040	−0.440591	−0.942466	0.175*	
H12B	0.914541	−0.400186	−0.986975	0.175*	
C13	1.0808 (13)	−0.2527 (12)	−0.9718 (4)	0.145 (2)	
H13A	1.161245	−0.293458	−0.993055	0.218*	
H13B	1.024480	−0.175271	−0.987935	0.218*	
H13C	1.133662	−0.215559	−0.943555	0.218*	
C2	−1.3727 (13)	−0.2480 (11)	−0.5256 (4)	0.139 (3)	
H2A	−1.381933	−0.323663	−0.501410	0.167*	
H2B	−1.477161	−0.237448	−0.541630	0.167*	
C1	−1.3215 (13)	−0.1070 (10)	−0.5029 (4)	0.139 (3)	
H1A	−1.401592	−0.077904	−0.479592	0.209*	
H1B	−1.217725	−0.119369	−0.487328	0.209*	
H1C	−1.312388	−0.033665	−0.527309	0.209*	

### Atomic displacement parameters (Å^2^)

**Table:**

	*U*^11^	*U*^22^	*U*^33^	*U*^12^	*U*^13^	*U*^23^
O6	0.080 (3)	0.089 (3)	0.089 (3)	0.000 (2)	0.019 (3)	−0.013 (2)
O3	0.069 (3)	0.093 (3)	0.082 (3)	−0.014 (2)	0.010 (2)	−0.004 (2)
N2	0.066 (3)	0.055 (2)	0.076 (3)	0.001 (2)	−0.002 (3)	−0.001 (2)
O2	0.077 (3)	0.097 (3)	0.117 (4)	0.014 (3)	0.032 (3)	0.019 (3)
N1	0.063 (3)	0.063 (2)	0.076 (3)	0.001 (2)	0.005 (3)	0.000 (2)
O4	0.132 (5)	0.109 (4)	0.124 (4)	0.004 (4)	0.050 (4)	−0.017 (3)
O5	0.118 (4)	0.160 (4)	0.159 (5)	−0.008 (3)	0.057 (4)	−0.021 (4)
O1	0.091 (4)	0.144 (4)	0.133 (5)	−0.035 (4)	0.027 (4)	0.004 (4)
C19	0.060 (3)	0.051 (2)	0.065 (3)	0.004 (2)	−0.002 (3)	0.002 (2)
C8	0.059 (3)	0.054 (2)	0.064 (3)	0.003 (2)	−0.005 (3)	0.004 (2)
C3	0.071 (5)	0.096 (5)	0.102 (5)	−0.003 (4)	−0.002 (4)	0.034 (4)
C5	0.056 (3)	0.071 (3)	0.071 (4)	0.003 (3)	0.004 (3)	0.008 (3)
C16	0.068 (3)	0.063 (3)	0.066 (4)	0.002 (3)	0.002 (3)	−0.001 (3)
C11	0.066 (4)	0.052 (2)	0.067 (3)	0.005 (2)	−0.010 (3)	0.004 (2)
C9	0.064 (4)	0.064 (3)	0.079 (4)	−0.005 (3)	−0.006 (3)	−0.008 (3)
C18	0.062 (3)	0.056 (3)	0.078 (4)	0.006 (2)	0.001 (3)	−0.005 (3)
C17	0.064 (3)	0.063 (3)	0.077 (4)	−0.002 (3)	−0.004 (3)	−0.003 (3)
C10	0.061 (3)	0.064 (3)	0.078 (4)	−0.016 (3)	−0.001 (3)	−0.007 (3)
C22	0.062 (3)	0.047 (2)	0.076 (4)	−0.002 (2)	−0.002 (3)	−0.003 (2)
C20	0.075 (4)	0.056 (3)	0.082 (4)	−0.006 (3)	−0.001 (4)	−0.001 (3)
C6	0.075 (4)	0.069 (3)	0.085 (4)	−0.003 (3)	0.002 (4)	−0.018 (3)
C21	0.082 (4)	0.062 (3)	0.083 (4)	−0.006 (3)	0.009 (4)	−0.015 (3)
C4	0.079 (5)	0.099 (4)	0.079 (4)	−0.018 (4)	0.004 (4)	0.007 (4)
C7	0.064 (4)	0.060 (3)	0.089 (4)	−0.007 (3)	−0.007 (4)	−0.004 (3)
C14	0.082 (5)	0.089 (4)	0.083 (5)	0.011 (4)	0.017 (4)	−0.004 (4)
C15	0.075 (4)	0.082 (3)	0.078 (4)	−0.001 (3)	0.008 (4)	0.006 (3)
C12	0.118 (4)	0.160 (4)	0.159 (5)	−0.008 (3)	0.057 (4)	−0.021 (4)
C13	0.118 (4)	0.160 (4)	0.159 (5)	−0.008 (3)	0.057 (4)	−0.021 (4)
C2	0.121 (5)	0.146 (5)	0.151 (6)	0.013 (5)	0.061 (5)	0.007 (5)
C1	0.121 (5)	0.146 (5)	0.151 (6)	0.013 (5)	0.061 (5)	0.007 (5)

### Geometric parameters (Å, º)

**Table:**

O6—C16	1.355 (7)	C18—H18	0.9300
O6—C15	1.422 (8)	C17—H17	0.9300
O3—C5	1.367 (7)	C10—H10	0.9300
O3—C4	1.432 (8)	C22—H22	0.9300
N2—C22	1.275 (7)	C20—C21	1.372 (9)
N2—N1	1.419 (7)	C20—H20	0.9300
O2—C3	1.282 (9)	C6—C7	1.359 (9)
O2—C2	1.497 (10)	C6—H6	0.9300
N1—C11	1.274 (7)	C21—H21	0.9300
O4—C14	1.212 (8)	C4—H4A	0.9700
O5—C14	1.306 (9)	C4—H4B	0.9700
O5—C12	1.477 (10)	C7—H7	0.9300
O1—C3	1.213 (8)	C14—C15	1.474 (9)
C19—C18	1.378 (7)	C15—H15A	0.9700
C19—C20	1.403 (8)	C15—H15B	0.9700
C19—C22	1.463 (8)	C12—C13	1.415 (10)
C8—C7	1.382 (8)	C12—H12A	0.9700
C8—C9	1.406 (8)	C12—H12B	0.9700
C8—C11	1.431 (8)	C13—H13A	0.9600
C3—C4	1.498 (10)	C13—H13B	0.9600
C5—C10	1.375 (8)	C13—H13C	0.9600
C5—C6	1.407 (8)	C2—C1	1.502 (10)
C16—C17	1.375 (8)	C2—H2A	0.9700
C16—C21	1.379 (8)	C2—H2B	0.9700
C11—H11	0.9300	C1—H1A	0.9600
C9—C10	1.376 (8)	C1—H1B	0.9600
C9—H9	0.9300	C1—H1C	0.9600
C18—C17	1.385 (8)		
			
C16—O6—C15	118.7 (5)	C20—C21—C16	120.4 (5)
C5—O3—C4	116.0 (5)	C20—C21—H21	119.8
C22—N2—N1	111.5 (5)	C16—C21—H21	119.8
C3—O2—C2	114.9 (6)	O3—C4—C3	111.2 (6)
C11—N1—N2	112.6 (4)	O3—C4—H4A	109.4
C14—O5—C12	112.0 (7)	C3—C4—H4A	109.4
C18—C19—C20	118.5 (5)	O3—C4—H4B	109.4
C18—C19—C22	119.3 (5)	C3—C4—H4B	109.4
C20—C19—C22	122.2 (5)	H4A—C4—H4B	108.0
C7—C8—C9	117.4 (6)	C6—C7—C8	121.6 (6)
C7—C8—C11	124.6 (5)	C6—C7—H7	119.2
C9—C8—C11	118.0 (5)	C8—C7—H7	119.2
O1—C3—O2	125.4 (8)	O4—C14—O5	122.6 (7)
O1—C3—C4	119.2 (8)	O4—C14—C15	125.5 (7)
O2—C3—C4	115.4 (6)	O5—C14—C15	111.9 (6)
O3—C5—C10	125.5 (5)	O6—C15—C14	107.8 (6)
O3—C5—C6	115.3 (5)	O6—C15—H15A	110.1
C10—C5—C6	119.2 (6)	C14—C15—H15A	110.1
O6—C16—C17	124.4 (6)	O6—C15—H15B	110.1
O6—C16—C21	115.1 (5)	C14—C15—H15B	110.1
C17—C16—C21	120.5 (6)	H15A—C15—H15B	108.5
N1—C11—C8	124.2 (5)	C13—C12—O5	109.2 (8)
N1—C11—H11	117.9	C13—C12—H12A	109.8
C8—C11—H11	117.9	O5—C12—H12A	109.8
C10—C9—C8	121.7 (5)	C13—C12—H12B	109.8
C10—C9—H9	119.1	O5—C12—H12B	109.8
C8—C9—H9	119.1	H12A—C12—H12B	108.3
C19—C18—C17	121.6 (5)	C12—C13—H13A	109.5
C19—C18—H18	119.2	C12—C13—H13B	109.5
C17—C18—H18	119.2	H13A—C13—H13B	109.5
C16—C17—C18	119.0 (6)	C12—C13—H13C	109.5
C16—C17—H17	120.5	H13A—C13—H13C	109.5
C18—C17—H17	120.5	H13B—C13—H13C	109.5
C5—C10—C9	119.7 (5)	O2—C2—C1	105.5 (8)
C5—C10—H10	120.2	O2—C2—H2A	110.6
C9—C10—H10	120.2	C1—C2—H2A	110.6
N2—C22—C19	122.0 (5)	O2—C2—H2B	110.6
N2—C22—H22	119.0	C1—C2—H2B	110.6
C19—C22—H22	119.0	H2A—C2—H2B	108.8
C21—C20—C19	120.0 (6)	C2—C1—H1A	109.5
C21—C20—H20	120.0	C2—C1—H1B	109.5
C19—C20—H20	120.0	H1A—C1—H1B	109.5
C7—C6—C5	120.4 (5)	C2—C1—H1C	109.5
C7—C6—H6	119.8	H1A—C1—H1C	109.5
C5—C6—H6	119.8	H1B—C1—H1C	109.5
			
C22—N2—N1—C11	−177.6 (5)	C20—C19—C22—N2	−7.5 (8)
C2—O2—C3—O1	0.7 (11)	C18—C19—C20—C21	1.1 (8)
C2—O2—C3—C4	−179.3 (7)	C22—C19—C20—C21	−177.9 (6)
C4—O3—C5—C10	0.8 (9)	O3—C5—C6—C7	−177.8 (6)
C4—O3—C5—C6	179.7 (5)	C10—C5—C6—C7	1.2 (9)
C15—O6—C16—C17	−1.0 (9)	C19—C20—C21—C16	−2.3 (9)
C15—O6—C16—C21	179.3 (5)	O6—C16—C21—C20	−178.5 (6)
N2—N1—C11—C8	179.8 (5)	C17—C16—C21—C20	1.8 (9)
C7—C8—C11—N1	3.4 (9)	C5—O3—C4—C3	176.2 (5)
C9—C8—C11—N1	−176.4 (5)	O1—C3—C4—O3	−171.2 (6)
C7—C8—C9—C10	−0.4 (8)	O2—C3—C4—O3	8.8 (9)
C11—C8—C9—C10	179.4 (5)	C5—C6—C7—C8	0.0 (10)
C20—C19—C18—C17	0.6 (8)	C9—C8—C7—C6	−0.4 (9)
C22—C19—C18—C17	179.6 (5)	C11—C8—C7—C6	179.8 (5)
O6—C16—C17—C18	−179.8 (5)	C12—O5—C14—O4	−5.3 (13)
C21—C16—C17—C18	−0.1 (9)	C12—O5—C14—C15	174.9 (8)
C19—C18—C17—C16	−1.1 (9)	C16—O6—C15—C14	−178.8 (5)
O3—C5—C10—C9	176.9 (6)	O4—C14—C15—O6	−1.8 (11)
C6—C5—C10—C9	−2.0 (9)	O5—C14—C15—O6	178.0 (6)
C8—C9—C10—C5	1.6 (9)	C14—O5—C12—C13	165.9 (10)
N1—N2—C22—C19	−178.9 (5)	C3—O2—C2—C1	176.1 (8)
C18—C19—C22—N2	173.6 (5)		

### Hydrogen-bond geometry (Å, º)

**Table:**

*D*—H···*A*	*D*—H	H···*A*	*D*···*A*	*D*—H···*A*
C6—H6···O4^i^	0.93	2.57	3.483 (9)	169
C4—H4*B*···N1^ii^	0.97	2.69	3.618 (10)	161

Symmetry codes: (i) −*x*, *y*+1/2, −*z*−3/2; (ii) *x*−1, *y*, *z*.
